# Hemoptysis of Uncertain Cause Leading to Delayed Diagnosis in an Elderly Lady With Anti-glomerular Basement Membrane Disease

**DOI:** 10.7759/cureus.47917

**Published:** 2023-10-29

**Authors:** Anjana Razik, Zaheer Aslam

**Affiliations:** 1 General Internal Medicine, Bedford Hospital NHS Trust, Bedford, GBR; 2 Respiratory Medicine, Bedford Hospital NHS Trust, Bedford, GBR

**Keywords:** acute kidney injury, alveolar hemorrhage, hemoptysis, anti-glomerular basement membrane disease, goodpasture syndrome

## Abstract

Anti-glomerular basement membrane (GBM) disease or Goodpasture syndrome is a rare disorder characterized by anti-GBM autoantibodies targeting the type 4 collagen of the basement membrane, resulting in rapidly progressive glomerulonephritis with or without alveolar hemorrhage. Pulmonary manifestations are less common in the elderly. Isolated pulmonary manifestations are rare in all age groups, and even more so in the elderly. We present the case of a lady in her late 70s, who presented initially with massive hemoptysis in the absence of renal dysfunction, which was presumed to be secondary to underlying bronchiectasis and infection. However, she later developed rapidly progressive acute kidney injury despite improvement in pulmonary symptoms and was diagnosed with anti-GBM disease. The delay in diagnosis and subsequent treatment due to the atypical presentation resulted in irreversible renal injury and the need for lifelong dialysis. This case demonstrates the need to consider atypical presentations of rare disorders, to ensure early diagnosis and optimal prognosis, especially when the clinical history cannot be explained by findings on examination and investigation.

## Introduction

Anti-glomerular basement membrane (GBM) disease (previously known as Goodpasture syndrome) is a rare autoimmune small vessel vasculitis. It is characterized by the development of anti-GBM autoantibodies resulting in the deposition of antigen-antibody complexes along the basement membrane and consequent complement-mediated tissue injury [1]. It is unclear as to what triggers the development of anti-GBM antibodies.

The anti-GBM antibody targets the type 4 collagen in the GBM. The primary clinical manifestation is rapidly progressive (crescentic) glomerulonephritis [2]. The alveolar basement membrane also expresses type 4 collagen. However, in a healthy person, the alveolar endothelium acts as a protective barrier. Pulmonary manifestation in the form of alveolar hemorrhage usually occurs only when the permeability of the alveolar capillaries is heightened following an insult or due to a preexisting disease [3]. Pulmonary involvement is seen in up to two-thirds of cases, and it may be the predominant presentation in nearly half [4]. Patients may also present with pulmonary symptoms initially and develop renal failure shortly after. Thus, the clinical presentation is characterized by definite renal involvement, which may or may not be accompanied by pulmonary symptoms. Isolated pulmonary involvement is rare [5].

Here, we present the unusual case of a white woman in her late 70s. She presented initially with isolated pulmonary symptoms, which improved subsequently. However, she was later diagnosed with anti-GBM disease after she developed renal failure a few months later despite improvement in pulmonary symptoms.

## Case presentation

Ms. X, a white woman in her late 70s, was referred to us by her general practitioner (GP) following an episode of massive hemoptysis while on holiday. She had been seen by the GP multiple times previously with complaints of productive cough and copious amounts of sputum and was on treatment for a diagnosis of bronchiectasis. She was also on treatment for hypertension (on losartan) and dyslipidemia (on pravastatin) in addition to having cystocele, varicose veins, and no vision in the right eye. She had previously undergone cholecystectomy and hysterectomy and was a non-smoker.

**Figure 1:**
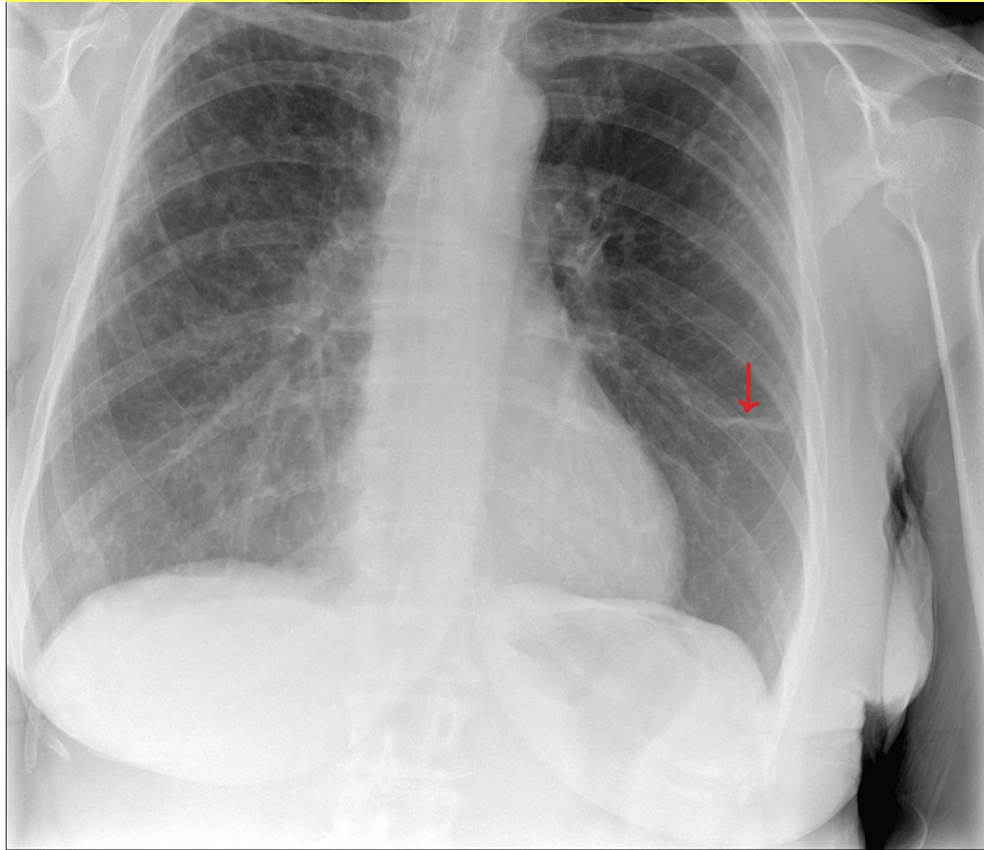
Chest X-ray showing bilateral abnormal shadowing Minimal abnormal shadowing can be seen in the right mid- and upper zones and the left mid-zone. The red arrow points to a small area of atelectasis in the left lower zone.

Chest X-ray done by the GP showed minimal abnormal shadowing throughout the right mid and upper zones and in the left mid zone and a small area of plate atelectasis in the left lower zone (Figure 1).

**Figure 2:**
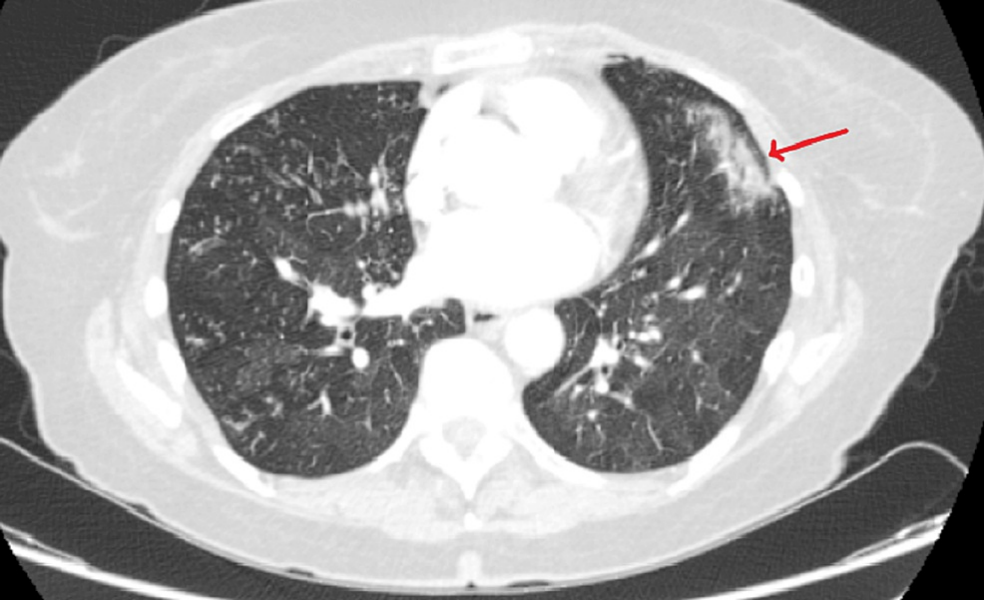
Axial CT pulmonary angiography slice showing non-specific changes The red arrow indicates the ground-glass opacity.

At the accident and emergency department at our hospital, she was suspected to have pulmonary embolism (PE). However, a CT pulmonary angiography showed no PE but revealed bilateral ill-defined pulmonary nodules, which aggregated to form focal areas of consolidation. There was also widespread bilateral tree-in-bud pattern and nonspecific ground glass changes (Figure 2). As blood investigations, including coagulation profile and creatinine levels, were unremarkable, she was tentatively diagnosed with bronchopneumonia, treated for the same, and transferred to the chest clinic.

Subsequently, she was followed up by the GP. Sputum culture two weeks later showed mixed growth, including Candida.

A month later at the chest clinic, she still had a cough, but no hemoptysis, and auscultation of the chest revealed a few crackles. There was no clubbing, blood pressure was 160/92, heart rate was 47 beats/min, and oxygen saturation was 96% on room air. The rest of the examination was unremarkable. She was treated symptomatically with a combination inhaler (beclomethasone + formoterol), a short course of oral prednisolone, and linctus codeine and posted for bronchoscopy three weeks later. Blood investigations performed during this visit, including renal function parameters, were within normal limits.

The bronchoscopy was uneventful. Bronchoalveolar lavage was collected for culture and cytology, and a biopsy was taken for histopathology. Culture of the bronchoalveolar lavage yielded mixed growth, which included Candida and Pseudomonas, and she was prescribed ciprofloxacin 750 mg twice a day for two weeks.

Cytology demonstrated oral squamous cells, bronchial epithelial cells, pulmonary macrophages, polymorphs, and bacteria. No malignant cells were seen.

Histopathology revealed benign respiratory lining epithelium with underlying seromucinous glands and connective tissue without evidence of acute inflammation, granulomata, atypia, dysplasia, features of invasive malignancy, or any other abnormalities.

No acid-fast bacilli were isolated from fluid culture even after six weeks of incubation.

**Figure 3:**
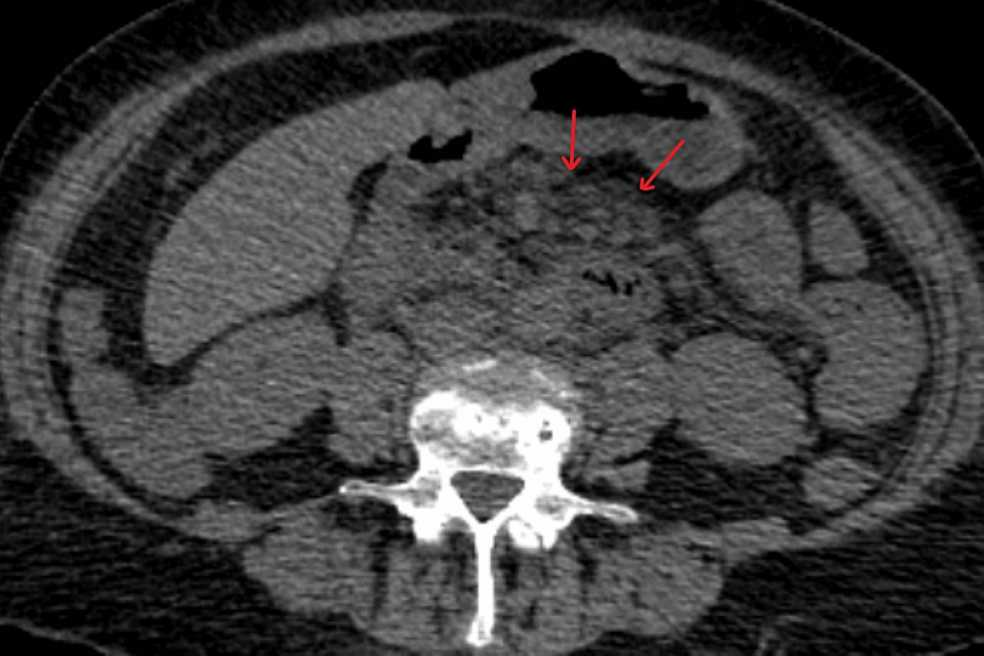
Non-contrast axial CT abdomen slice at the level of the root of mesentery suggestive of mesenteric panniculitis The red arrows point to an area of fat stranding and nodularity indicating focal extensive inflammatory changes around the root of mesentery

A follow-up CT thorax at three months after the initial presentation of massive hemoptysis showed significant improvements in pulmonary nodules. There was no indication of any new abnormalities. In view of the residual nodularity, she was advised a follow-up CT six months later. However, two weeks later, she presented with poor oral intake, intermittent loose stools, and abdominal pain, which had not resolved following treatment for a suspected urinary tract infection by the GP. Investigations showed a creatinine level of 923 µmol/L, urea of 34.2 mmol/L, serum amylase of 182 U/L, and CRP of 142 mg/L, and arterial blood gas analysis showed metabolic acidosis. A differential diagnosis of diverticulitis vs obstructive uropathy was made. However, CT KUB (kidneys, ureters, and bladder) showed no evidence of calculus or obstructive uropathy, while CT abdomen and pelvis revealed upper abdominal mesenteric inflammatory changes in the peripancreatic porta hepatis and at the root of the mesentery of the small bowel with few reactive lymph nodes, which was suggestive of mesenteric panniculitis, possibly associated with pancreatitis (Figure 3). IgG, IgA, IgM, complement C3, and C4 levels were within normal limits, and no paraproteins were detected on electrophoresis. ANA, ANCA, and EliA Symphony ENA screens were negative.

Despite fluid resuscitation, she became oligoanuric at around 5 mL/hr and was admitted to the critical care complex with Stage 3 acute kidney injury (AKI) for continuous veno-venous hemofiltration (CVVHF) prior to being transferred to a tertiary care center for further management and dialysis.

At the tertiary care center, anti-GBM antibodies were detected as part of the pre-dialysis screen, and she was diagnosed to have anti-GBM disease.

During a follow-up at the chest clinic a month later, she was clinically stable, was on dialysis thrice weekly and prophylactic azithromycin three times weekly, and had no respiratory issues.

She continues to come for six monthly follow-ups at the chest clinic. Bilateral bronchiectasis is clinically and radiologically stable. She is continuing dialysis three times a week.

## Discussion

Anti-GBM disease in itself is a rare disease with an estimated prevalence of less than one in a million [6]. The initial presentation of this case, with isolated pulmonary manifestation, is even more unusual. Very few cases of isolated pulmonary manifestations in anti-GBM have been reported to date, out of which none have been in elderly individuals [4,7,8]. In fact, elderly individuals have been reported to have fewer pulmonary manifestations [9,10].

Although we assumed that the massive hemoptysis in Ms. X at the initial presentation was secondary to an infective pathology, the bronchiectatic changes in subsequent CT scans were noted to be mild and unlikely to have caused hemoptysis to the described extent. Other major differential diagnoses of massive hemoptysis, including coagulopathies, PE, neoplasia, and pulmonary tuberculosis, had also been ruled out. Retrospectively, it would appear that the massive hemoptysis was due to anti-GBM disease. The nonspecific ground glass changes noted on the CT pulmonary angiography at presentation had probably been due to alveolar hemorrhage [11]. Unfortunately, at that point, we did not consider anti-GBM disease in the differential diagnoses as she had no renal symptoms and the renal function parameters were normal, in addition to the fact that anti-GBM itself is a rare disease, with isolated pulmonary symptoms being even more rare.

The management of anti-GBM disease involves immunosuppressants and plasmapheresis to remove autoantibodies [12]. It is possible that the improvement of pulmonary disease in Ms. X was mediated by the steroids that were prescribed for control of her wheeze. It is also possible that the anti-GBM disease was unmasked and the renal injury occurred when the steroids were stopped.

Most researchers maintain that it is unclear as to what triggers anti-GBM disease. However, some have postulated that pulmonary insults, such as an infection, smoking, inhaled hydrocarbons, and renal injury could be triggering factors [12,13]. The underlying bronchiectasis may have triggered the disease of Ms. X.

Although renal failure is a common complication in patients with anti-GBM disease, only a third of them end up requiring lifelong dialysis [14]. Renal damage is usually milder in the elderly [10]. Prompt diagnosis and early initiation of treatment help in limiting renal injury and ensuring a good prognosis. Unfortunately, Ms. X had an unusual presentation with isolated pulmonary symptoms in old age. The coexistence of a known pulmonary pathology (bronchiectasis) added to the complexity and subsequent delay in diagnosis, resulting in a poor prognosis.

## Conclusions

Anti-GBM disease is a rare entity in itself, and the presence of isolated pulmonary manifestations is even rarer. When a patient presents with symptoms suggestive of alveolar hemorrhage, and common etiologies have been ruled out, it is important to consider and investigate for less common etiologies, especially when the clinical history cannot be explained by findings on examination and investigation. A vasculitis screen must also be considered for all patients presenting with hemoptysis. This is particularly relevant in anti-GBM disease where early diagnosis and prompt initiation of appropriate treatment can avoid the need for lifelong dialysis, thereby significantly improving the prognosis.
